# Rare cause of symptoms: Cardiac fibroma in an adult

**DOI:** 10.1016/j.xjtc.2026.102511

**Published:** 2026-07-15

**Authors:** Philipp Witte, Julian D. Widder, Anne K.E. Bruening, Reinhard Zabel, René Schramm, Julia Goette, Jan Rabe, Raihanatou Danebrock, André Renner, Jan Gummert, Michiel Morshuis, Sabina P.W. Guenther

**Affiliations:** a Department of Thoracic and Cardiovascular Surgery, Heart and Diabetes Center NRW, Ruhr-University Bochum, Bad Oeynhausen, Germany; b Medical Clinic IV - Department of Cardiology, Angiology and Intensive Care, Municipal Hospital Karlsruhe, Karlsruhe, Germany; c Institute of Radiology, Nuclear Medicine and Molecular Imaging, Heart and Diabetes Center NRW, Ruhr-University Bochum, Bad Oeynhausen, Germany; d Institute for Diagnostic and Interventional Radiology, Municipal Hospital Karlsruhe, Karlsruhe, Germany; e Institute for Pathology, Johannes Wesling University Hospital, Ruhr-University Bochum, Minden, Germany

**Figure:**
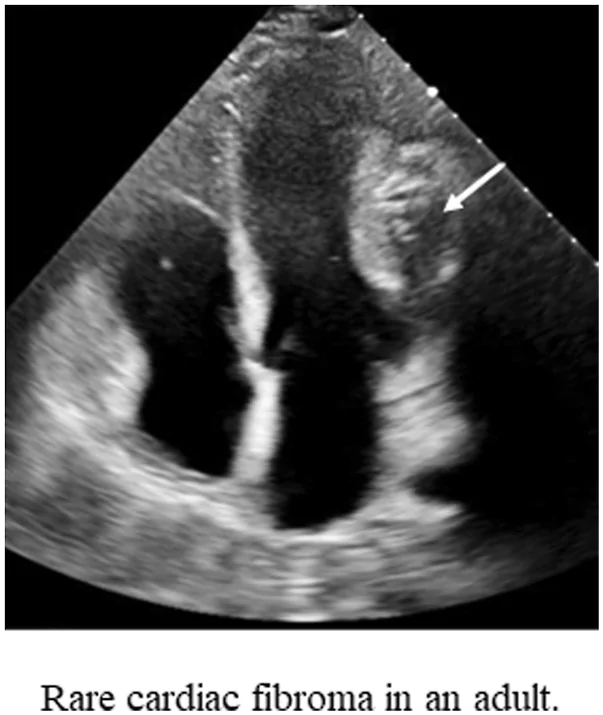
A rare cardiac fibroma in an adult is shown.

**Central Message**

Cardiac fibromas are rare benign tumors predominantly diagnosed in children and seldomly in adults. Multimodal imaging is key. Complete resection is technically feasible and considered curative.

Cardiac fibromas are rare benign tumors typically occurring in children, with limited data available in adults.^1^^,^^2^ Diagnostic and treatment guidelines are lacking, but surgical resection is considered the primary treatment.^1^ However, guidance on surgical strategies is scarce.^3^

## Case Report

### Patient History

The patient provided consent for the publication of her information. A 35-year-old woman presented with chest pain. Comorbidities included hypertension, smoking, and obesity. On clinical examination, her cardiopulmonary status was unremarkable.

Laboratory tests revealed elevated high-sensitivity troponin I (123 ng/L; reference range <2 ng/L), N-terminal pro B-type natriuretic peptide (1432 pg/mL; <125 pg/mL), d-dimers (0.68 μg/mL; <0.5 μg/mL), and leukocytes (12.12/nL; 4.0-10.0/nL). Electrocardiogram demonstrated nonspecific T-wave abnormalities. Echocardiography identified a 3- × 4-cm mass within the lateral left ventricular (LV) wall (Figure 1). LV function, the atria, right ventricle, and valves were normal.

**Figure 1:**
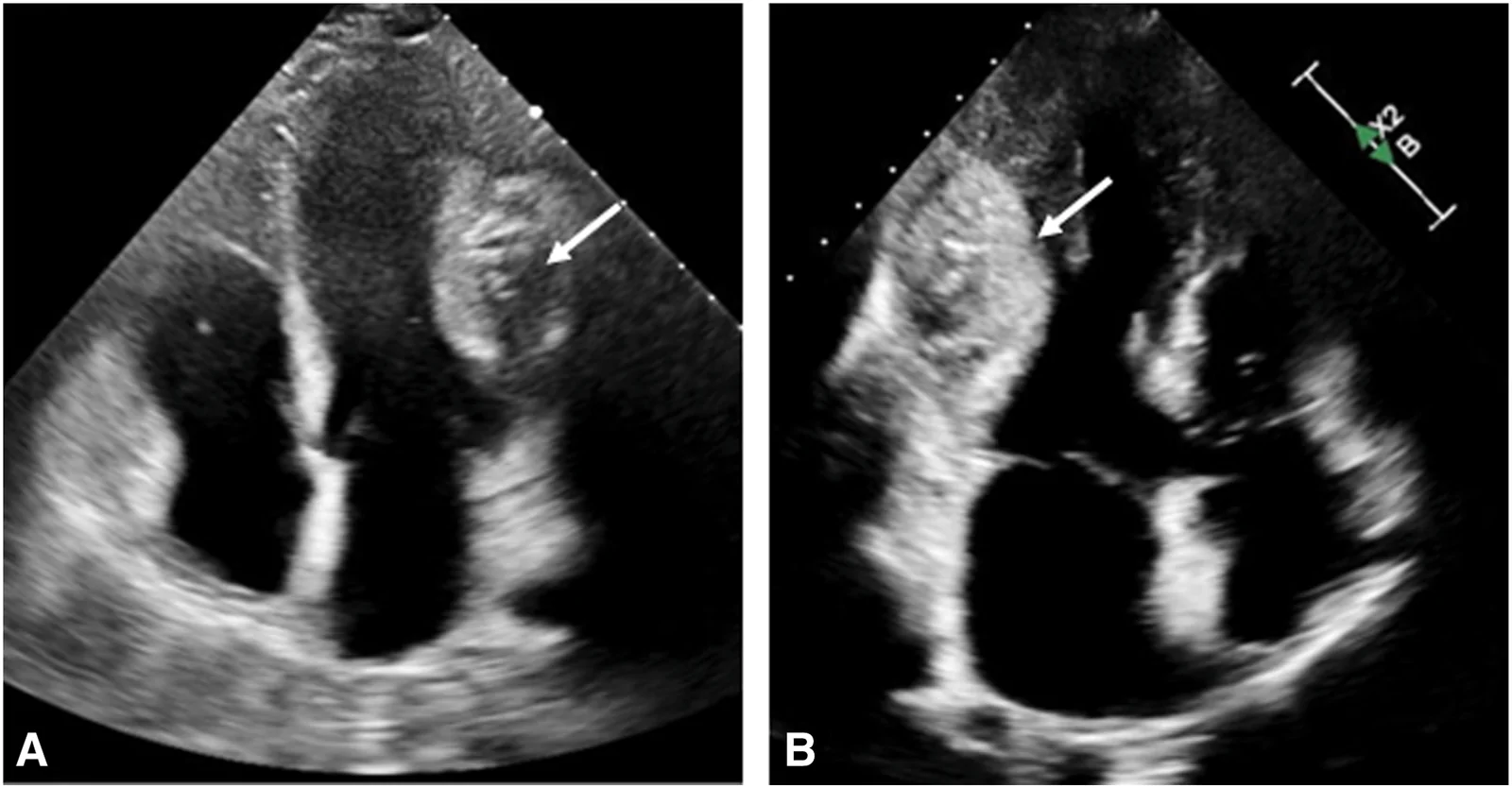
Transthoracic echocardiography in different projections (A, B). Lateral left ventricular wall tumor (*arrow*) with hyperechoic calcifications is shown.

Further evaluation included cardiac magnetic resonance imaging, which showed a 3.1- × 4.9- × 4.7-cm, well-delineated, intramural midmyocardial LV lateral wall mass protruding into the LV cavity. It was T1 iso- and T2 hypointense and lacked first-pass-perfusion uptake but showed marked late gadolinium enhancement, consistent with cardiac fibroma. The intramural mass itself was noncontractile; however, the adjacent layers of myocardium demonstrated preserved contractility. Computed tomography (CT) imaging excluded additional tumors or metastases but demonstrated mass calcifications. Fluorodeoxyglucose positron emission tomography imaging showed low uptake (Figure 2).

**Figure 2:**
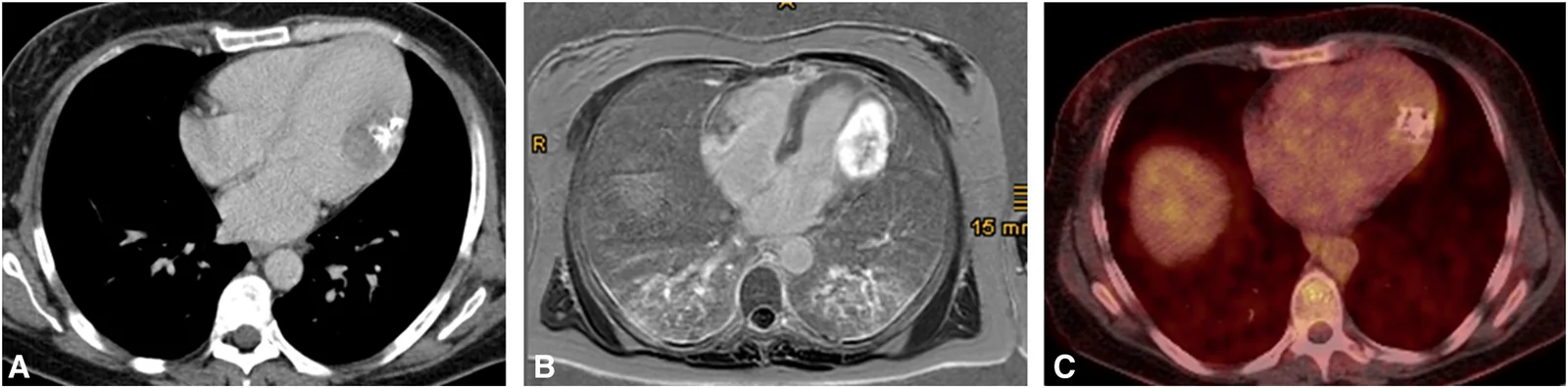
A, Computed tomography scan shows a intramural lateral left ventricular wall tumor with coarse calcifications. B, Magnetic resonance imaging scan shows that the mass lacked first-pass-perfusion uptake but strong late gadolinium enhancement. STIR sequences confirmed its intramurality. C, FDG-positron emission tomography/computed tomography imaging revealed low FDG tumor uptake (SUVmax 4.8). *STIR*, Short tau inversion recovery; *FDG*, fluorodeoxyglucose; *SUVmax*, maximum standardized update value.

Coronary angiography was used to assess the patient’s coronary status and tumor perfusion. It revealed single-vessel disease of the left anterior descending artery (Figure 3) with 2 lesions: a severe proximal stenosis due to fibromatous plaques confirmed by intravascular ultrasound and a severe midsegment stenosis caused by tumor compression. After heart-team discussion, percutaneous coronary intervention and drug-eluting stent implantation were performed (Figure 4), relieving the patient's symptoms. Troponin levels decreased after this intervention. As a result of the patient's clinical symptoms and uncertainty regarding the intraoperative anatomical conditions, preoperative percutaneous coronary intervention was preferred to potential surgical revascularization. The patient was subsequently referred for tumor resection.

**Figure 3:**
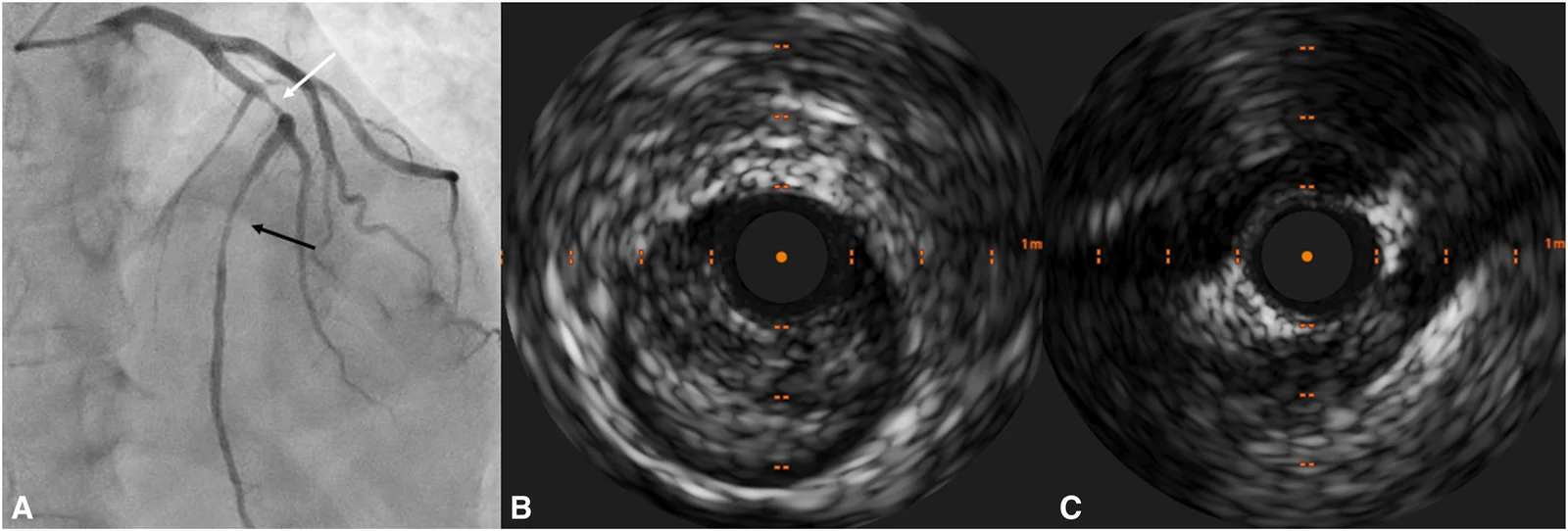
A, Coronary angiogram of the left anterior descending artery showed a proximal stenosis caused by fibromatous plaques (*white arrow*), and a midsegment stenosis caused by tumor compression (*black arrow*). B and C, Intravascular ultrasound shows proximal stenosis caused by fibromatous plaques and midsegment stenosis caused by tumor compression.

**Figure 4:**
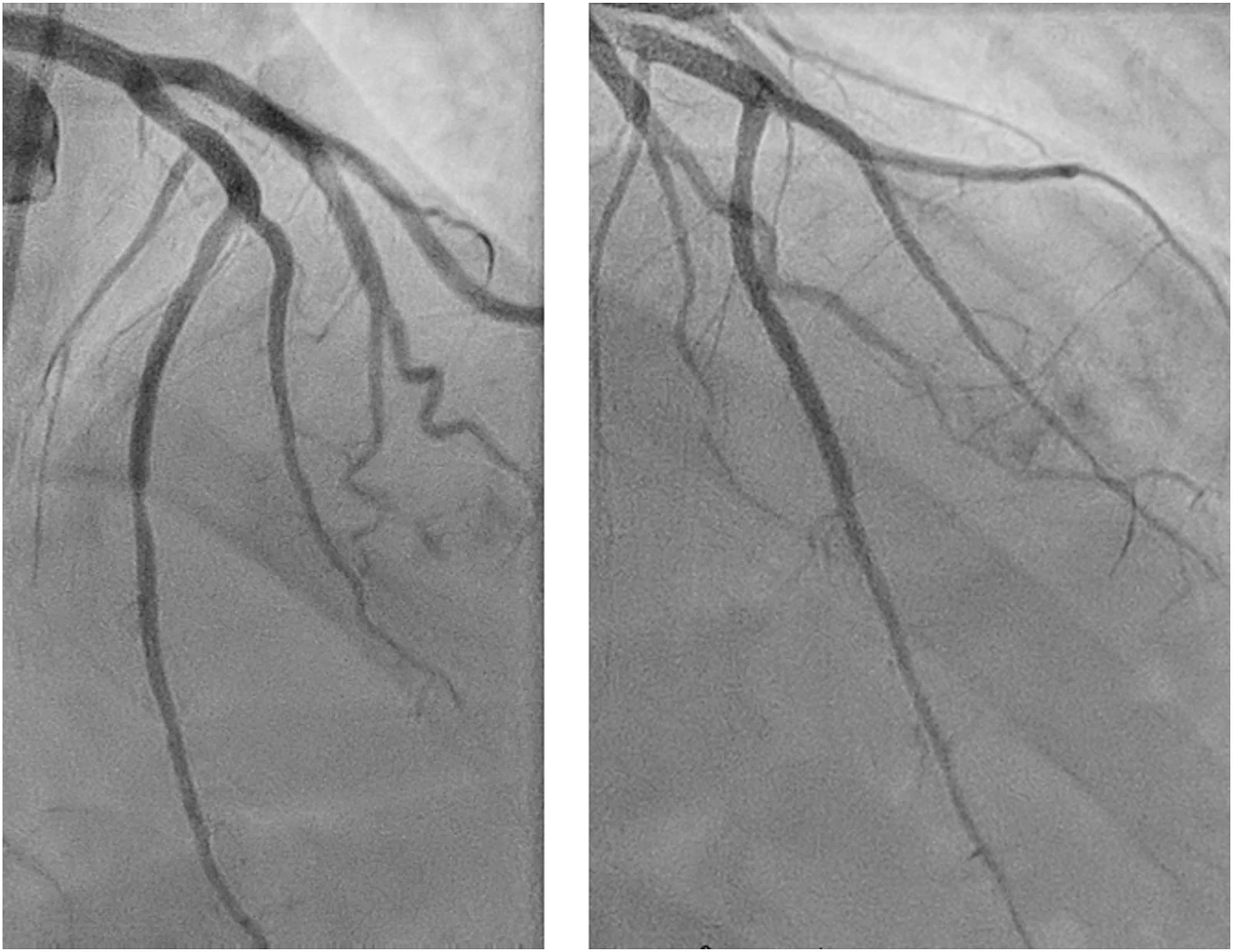
Coronary angiogram after percutaneous coronary intervention and stenting.

### Surgical Procedure

After median sternotomy, cardiopulmonary bypass was instituted via bicaval and aortic cannulation. Crossclamping and cardioplegia administration via the aortic root followed. The tumor was identified in the anterolateral LV wall. The left anterior descending and the left marginal ran close by. The tumor was well-delineated, allowing for dissection from the surrounding tissue using sharp and electrocautery dissection (Figure 5). In toto resection was successful. An LV wall defect (approximately 2.5 cm^2^) was repaired with a bovine pericardial patch (Peri-Guard, 6 cm × 8 cm; Baxter) using interrupted 4-0 polypropylene sutures, reinforced with felt pledgets. After reperfusion, weaning from cardiopulmonary bypass was uneventful.

**Figure 5:**
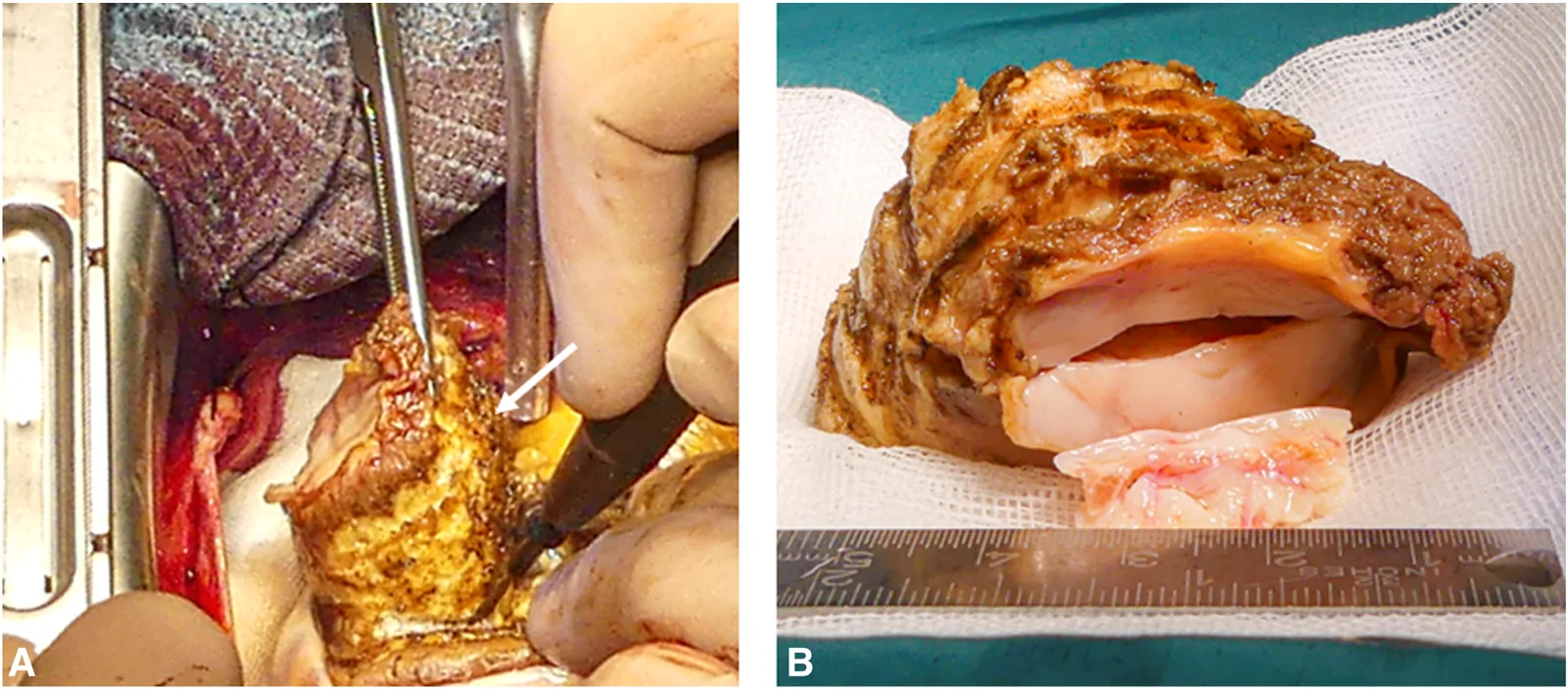
A, Surgical tumor (*arrow*) resection. B, Resected tumor (4.5 cm in length).

Histopathology demonstrated spindle cell proliferation (Figure E1). Conventional and immunohistochemical staining confirmed cardiac fibroma, without suspicion of malignancy.

The patient was discharged on postoperative day 8. Echocardiography showed normal LV dimensions and function. At 1 year postoperatively, she continues to be free of cardiovascular symptoms. Magnetic resonance imaging (MRI) performed 9 months postoperatively ruled out residual or recurrent tumor (Figure E2).

## Discussion

Two aspects of this case are noteworthy. First, the patient's age is unusual. Cardiac fibromas typically affect children. Rarely is de novo diagnosis made in adults.^1^ However, fibromas should be considered in the differential diagnosis of adult cardiac masses, alongside rhabdomyomas, myxomas, lipomas, and metastases.^1^^,^^4^

Second, the clinical presentation is interesting. Cardiac fibromas can be asymptomatic and incidentally diagnosed. Mass effects with cavity or outflow tract obstruction, valvular impairment, or conduction disturbances likely due to infiltration can cause symptoms, eg, dyspnea, arrhythmias, syncope, heart failure, or sudden cardiac death.^1^^,^^4^^,^^5^ Coronaries are typically unaffected. Rarely, as here, compression causes chest pain.

Accurate diagnosis is paramount. Diagnostic algorithms should commence with echocardiography, followed by MRI and/or CT. Cardiac fibromas are typically intramural, growing toward the chamber. The mass effect results in a distinct local thickening of the myocardial wall. Fibromas are noncontractile, but the adjacent layers of myocardium show preserved contractility. They appear well-defined; however, they may infiltrate the surrounding myocardium.^1^ Traditionally described as homogenous, frequently calcifications cause heterogenous appearances. Avascularity is characteristic. MRI is the preferred modality for differential diagnosis, typically demonstrating T1 iso- or slight hypointensity, T2 hypointensity, and marked late gadolinium enhancement.^1^ The latter indicates a significantly increased extracellular volume, ie, a high proportion of fibrous tissue (consistent with fibroma). Multimodal imaging, including cardiac CT, defines surgical anatomy and relationships to pertinent structures.^1^ Positron emission tomography imaging has lesser diagnostic value but may aid excluding differential diagnoses.

To prevent severe complications, complete resection is considered the preferred treatment.^2^^,^^3^ Alternatives include medical management (eg, for arrhythmias), rarely, partial resection if critical structure involvement prohibits complete excision, or transplantation. Surveillance may be considered in selected asymptomatic adults.^1^

Cardiac fibromas are typically firm, solitary lesions.^2^ Although benign, they pose surgical challenges if located adjacent to critical structures. Nevertheless, total resection is often feasible, yielding excellent results. The surgical armamentarium may require ventricular wall or septum reconstruction, valve repair, or bypass grafting. Fibromas lack encapsulation^2^^,^^5^ but in our experience permit secure dissection planes, enabling in toto resection with minimal collateral damage. The surrounding tissue quality is unaltered, facilitating reconstructive procedures.

## Conclusions

Cardiac fibromas are rare benign tumors that may cause unexpected symptoms. Predominantly diagnosed in children, they are rare in adults. Multimodal imaging is key. Complete resection is technically feasible and considered curative.

## Conflict of Interest Statement

The authors reported no conflicts of interest.

The *Journal* policy requires editors and reviewers to disclose conflicts of interest and to decline handling or reviewing manuscripts for which they may have a conflict of interest. The editors and reviewers of this article have no conflicts of interest.

## Figures and Tables

**Figure E1:**
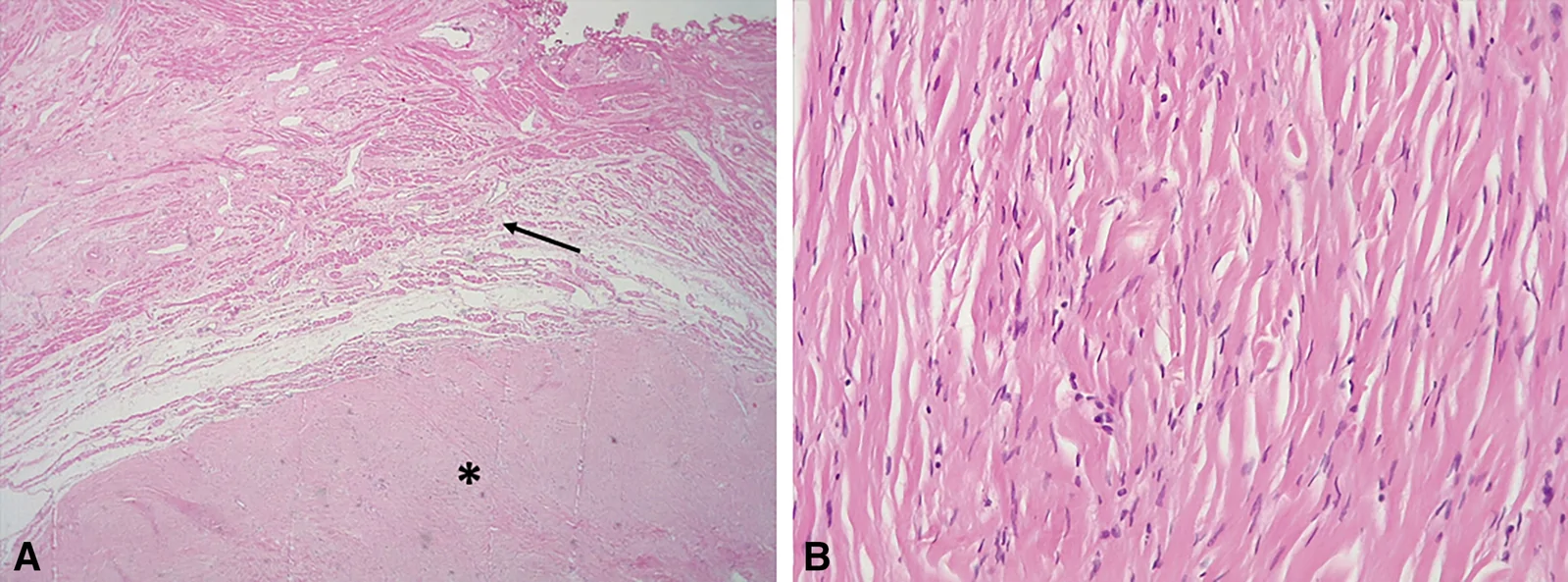
Histopathology. A, Hematoxylin and eosin staining (H&E), ×25. The tumor (*asterisk*) is well-delineated from the myocardium (*arrow*). B, H&E, ×200. Benign spindle-shaped tumor cells are shown.

**Figure E2:**
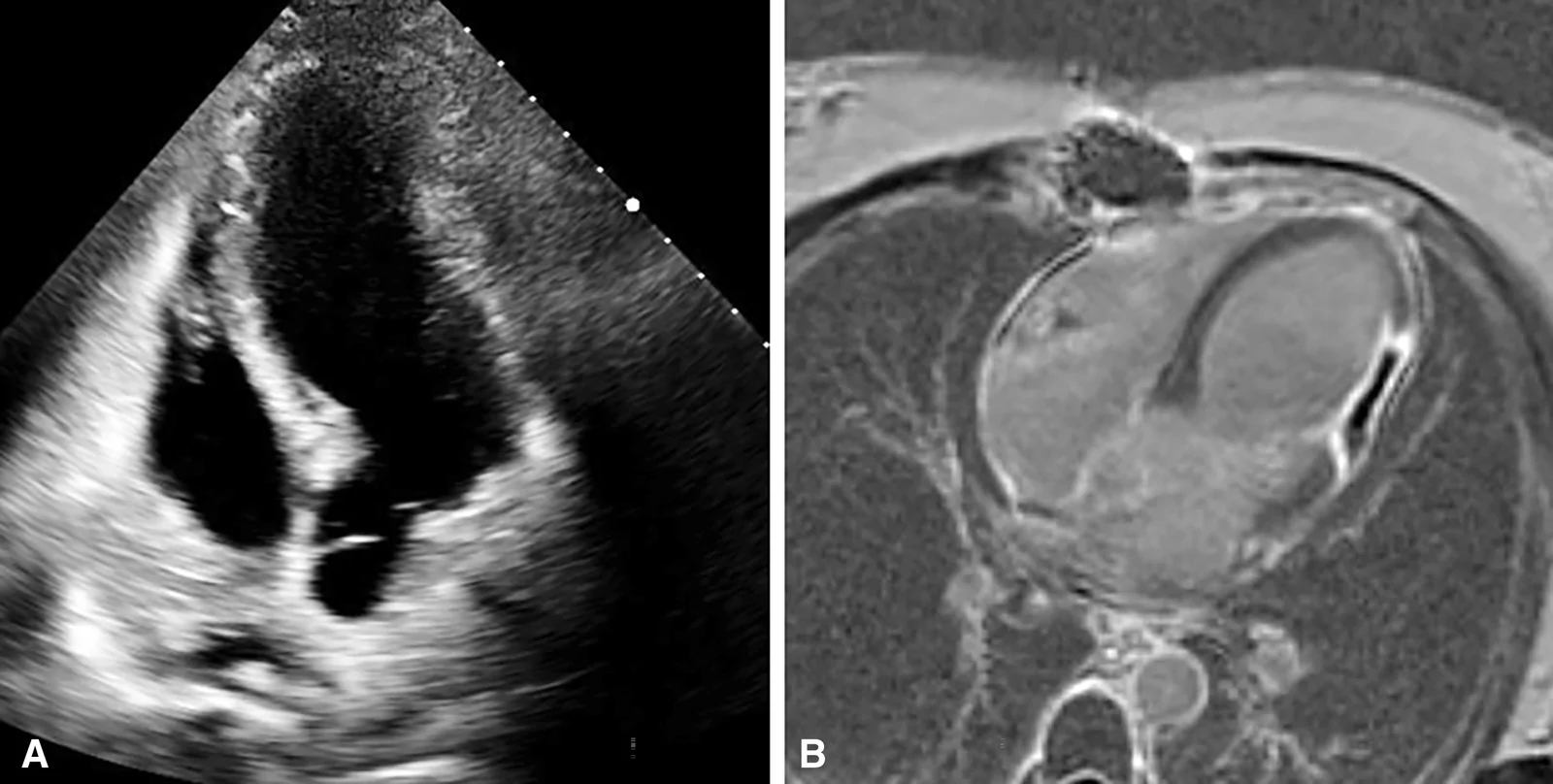
A, Echocardiography on postoperative day 7. B, Magnetic resonance imaging results 9 months later.
